# Challenges and Scientific Prospects of the Newest Generation of mRNA-Based Vaccines against SARS-CoV-2

**DOI:** 10.3390/life11090907

**Published:** 2021-08-31

**Authors:** Daniela Calina, Antonio F. Hernández, Thomas Hartung, Alexey M. Egorov, Boris Nikolaevich Izotov, Taxiarchis Konstantinos Nikolouzakis, Aristidis Tsatsakis, Panayiotis G. Vlachoyiannopoulos, Anca Oana Docea

**Affiliations:** 1 Department of Clinical Pharmacy, University of Medicine and Pharmacy of Craiova, 200349 Craiova, Romania; 2 Department of Legal Medicine and Toxicology, School of Medicine, University of Granada, 18016 Granada, Spain; ajerez@ugr.es; 3 Biomedical Research Institute of Granada ibs.GRANADA, Avda. de las Fuerzas Armadas, 2, 18014 Granada, Spain; 4 Consortium for Biomedical Research in Epidemiology & Public Health (CIBER en Epidemiología y Salud Pública), CIBERESP, Instituto de Salud Carlos III, Monforte de Lemos 3-5, Pabellón 11, Planta 0, 28029 Madrid, Spain; 5 CAAT-Europe, University of Konstanz, 78464 Konstanz, Germany; thartun1@jhu.edu; 6 CAAT, Bloomberg School of Public Health, Johns Hopkins University, Baltimore, MD 21205, USA; 7 Chumakov Federal Scientific Center for Research and Development of Immune and Biological Products, Russian Academy of Sciences, 108819 Moscow, Russia; alex.m.egorov@gmail.com; 8 Division of Medical Sciences, Russian Academy of Sciences, 119991 Moscow, Russia; 9 Department of Analytical and Forensic Medical Toxicology, Sechenov University, 119991 Moscow, Russia; ankaoana@yahoo.com (B.N.I.); tsatsaka@uoc.gr (A.T.); 10 Laboratory of Toxicology, Medical School, University of Crete, 70013 Heraklion, Greece; medp2011836@med.uoc.gr; 11 Department of Pathophysiology, School of Medicine, National and Kapodistrian University of Athens, 15772 Athens, Greece; pvlah@med.uoa.gr; 12 Department of Toxicology, University of Medicine and Pharmacy of Craiova, 200349 Craiova, Romania

**Keywords:** COVID-19 pandemic, public health, coronaviruses, mRNA vaccines, side effects

## Abstract

In the context of the current COVID-19 pandemic, traditional, complex and lengthy methods of vaccine development and production would not have been able to ensure proper management of this global public health crisis. Hence, a number of technologies have been developed for obtaining a vaccine quickly and ensuring a large scale production, such as mRNA-based vaccine platforms. The use of mRNA is not a new concept in vaccine development but has leveraged on previous knowledge and technology. The great number of human resources and capital investements for mRNA vaccine development, along with the experience gained from previous studies on infectious diseases, allowed COVID-19 mRNA vaccines to be developed, conditionally approved and commercialy available in less than one year, thanks to decades of basic research. This review critically presents and discusses the COVID-19 mRNA vaccine-induced immunity, and it summarizes the most common anaphylactic and autoimmune adverse effects that have been identified until now after massive vaccination campaigns.

## 1. Introduction

SARS-CoV-2 is an enveloped virus with a single-stranded RNA genome that belongs to the β-coronavirus family such that it is structurally and functionally similar to other members of this family, especially SARS-CoV (also called SARS-CoV-1) [1,2]. The structure, mode of infection, replicative cycle and type of induced immune response could therefore be anticipated based on previous knowledge [3].

The spike (S) glycoprotein in SARS-CoV-2 plays a pivotal role as a membrane fusion protein; it consists of two subunits with distinct functions: S1, which contains a receptor-binding domain (RBD) that recognizes and binds to the host cell receptor angiotensin-converting enzyme 2 (ACE2), and S2, which is essential for the virus–cell membrane fusion process. When the S protein binds to the ACE2 receptor, it is cleavaged by a serine protease located on the host cell membrane, thus promoting virus entry into the cell. Once the SARS-CoV-2 virus gains entry into the cell (initially airway epithelial cells), proinflammatory cytokines are released which can eventually trigger a cytokine storm, resulting in lung damage and augmented COVID-19 severity [4]. Patients infected with SARS-CoV-2 exhibit clinical manifestations ranging from nonspecific mild symptoms to severe pneumonia and damage of organ functions [5,6]. While the lung is the primary viral target, with a life-threatening acute respiratory distress syndrome (ARDS) as the COVID-19 signature, COVID-19 is not a respiratory illness alone [7]. The cardiovascular system, brain, kidney, liver and immune system are also affected by the infection [8].

Because the RNA sequence encoding S protein of SARS-CoV-2 is approximately 75% homologous to that of SARS-CoV virus, attachment of the S protein to ACE2 receptors, fusion of the viral envelope with the host cell membrane, and penetration of the virus into the cytoplasm occurs similarly for SARS-CoV and SARS-CoV-2 [9,10]. However, the immunodominant component of S protein, the RBD, is less conserved showing approximately 47% similarity between SARS-CoV and SARS-CoV-2 [1,11]. This knowledge allowed us, based on previous experience with SARS-CoV and other coronaviruses, to propose methods for developing distinct vaccines against COVID-19 that may be safe and effective at preventing serious illness, hospitalization and COVID-19-related deaths [12,13].

Diverse vaccine technology platforms have been developed for COVID-19, including nucleic acid (RNA and DNA), protein subunit, virus-like particles, inactivated virus, viral vectors and live attenuated virus [14,15]. The recent interest in mRNA vaccines has been boosted by technological developments that have enhanced mRNA stability and improved vaccine delivery (Borah et al., 2021). Ultimately, the development of mRNA vaccines did not start from scratch but was built on more than 30 years of experience of the scientific community aimed to develop injectable mRNA compounds [16]. The principles of messenger RNA (mRNA) vaccination techniques date back to the early 1990s [17], and dozens of studies on the subject have been published since then. During these three decades, significant progress has been made on how the mRNA molecule is constructed to be efficiently processed by cells, and how these molecules can be packaged to ensure protection from degradation on their way to target cells [18].

## 2. mRNA Vaccines: Head-to-Head Benchmarks

### 2.1. RNA: A Brief Overview and Issues Related to Its Stability

RNA molecules have multiple roles in all branches of the tree of life, from bacteria to mammals, and their synthesis and degradation are intensely controlled [19]. mRNA strands are large and negatively charged molecules, such that they cannot cross the lipid membrane of cells.

Moreover, mRNA is intrinsically unstable and prone to degradation by ribonucleases (RNases), which are widely distributed throughout all tissues and also present in the environment (e.g., bacteria, microorganisms, etc.). Storage at a low temperature reduces the chances that RNases, even if they have somehow contaminated the solution, degrade RNA [20]. An effective delivery of mRNA into target cells requires protection against the action of endogenous RNases, which can be conferred by using lipid nanoparticles (LNPs) as carriers of the mRNA [21]. The lipid coating also helps mRNA enter muscle and immunological cells near the vaccination sites. LNPs encapsulate mRNA and assemble it into the stable lipid bilayers, which are then ingested by cells through a variety of endocytosis pathways (Park et al., 2021). Once inside the cell, the molecule is more protected against the action of RNases compared to other mRNAs due to its modified nucleotide structure [22].

From the point of view of manufacturing technology, none of these lipids can be easily replaced because together they have several essential roles in obtaining vaccines with the new mRNA technology [23].

(i) Structural and protective: The aforementioned lipid compounds have a remarkable ability to self-assemble around mRNA [24] forming stable, spherical nanoparticles below 100 nm in diameter, approximately the average size of a SARS-CoV-2 virion. The trafficking of mRNA into such lipid spheres (LNP) protects it against enzymes that would otherwise digest it very quickly, such as RNases [25].

(ii) Role of “Trojan horse” for access inside cells [26]. On its own, mRNA would have much more difficulties penetrating inside cells because there is no “gate” for nucleic acids. With the incorporation of mRNA into LNPs that biochemically resemble the cell membrane, cells uptake LNPs through a process called endocytosis [27].

(iii) Adjuvant role, due to the immunogenic nature of lipids [28]. Lipids are adjuvants, perfectly capable of causing immune reactions themselves [29]. The purpose of the adjuvants is to help enhance the effect of the vaccine.

RNA is also more susceptible to spontaneous degradation in aqueous medium compared to DNA due to the presence of additional oxygen atoms in its composition. An additional factor would be the “unpacked”, accessible state in which these molecules are usually found. While DNA is usually found in “double-stranded” structures, consisting of two complementary DNA molecules, mRNA functions as a single molecule [30].

Under proper storage conditions (such as a solution with adequate pH and the absence of RNases), RNA can withstand a long time. Indeed, it can be stored for years at −80 °C without losses affecting use for research purposes [31]. Conversely, any deviation from the tested protocol under current real-life conditions may result in mRNA degradation that accounts for a possible decrease in the vaccine efficiency by decreasing the amount of mRNA available [30].

### 2.2. The New Concept and Development of mRNA Vaccines against SARS-CoV-2

**Figure 1:**
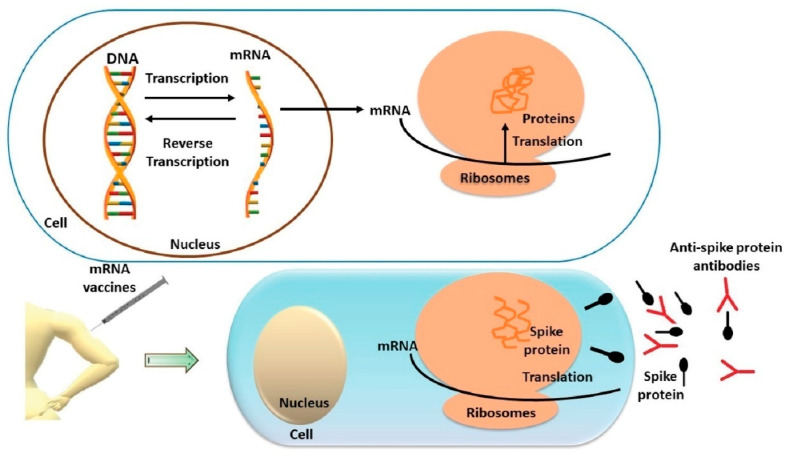
Diagram of the new concept of mRNA vaccines against SARS-CoV-2. Legend: deoxyribonucleic acid (DNA), messenger RNA (mRNA), ribonucleic acid (RNA).

The new generation and the new concept of mRNA vaccines against SARS-CoV-2 by-pass the transcription of DNA to mRNA in the nucleus. Instead, the mRNA is delivered directly to the cytoplasm where they promote a transient expression of S protein in the ribosomes lasting a few days (Figure 1). The mRNA vaccine does not encode the entire SARS-CoV-2 virus but only the S protein. The translation of the exogenous antigen is controlled by the lifetime of encoding mRNA, which in turn depends on the cellular degradation pathways [32].

The mRNA used in the technology of the new mRNA vaccines has some modifications (modRNA), in order to make the vaccine more efficient [16]. The main change is the replacement of the uracil nitrogen base (alternatively uridine triphosphate or U) with the functional isomer N1 methyl pseudouridine (ψ), which enhanced the translation of modRNA compared to mRNA [33]. Moreover, this substitution seems to reduce the rate of intracellular degradation, which is an advantage. It should be noted that ψ naturally occurs in RNA in almost every cell in our body [34].

The remarkable advances made in the biological stabilisation of mRNA, as well as the very encouraging results in animal models, allowed clinical trials to be initiated in 2010 [35]. A decade later, mRNA vaccines were tested in ten clinical trials for infectious diseases (HIV, Zika, influenza and rabies) and in more than fifty trials for various cancers [36]. Earlier vaccine research conducted by Moderna for Middle East Respiratory Syndrome coronavirus (MERS-CoV, 50% similar to SARS-CoV-2) led to solid preclinical efficacy and safety data on a potential candidate mRNA vaccine, thus making it possible to skip some steps for COVID-19 mRNA vaccine development [37]. In particular, traditional animal testing for both efficacy and safety testing was mostly skipped and some novel approaches were utilized [38]. For SARS-CoV-2, the process has taken less than one year instead of 10–15 years that the development of a vaccine normally takes [39]. In addition, logistical issues, especially manufacturing and distribution were certainly sped-up.

RNA platforms have raised huge interest because of their safety profile, higher efficacy, lower production cost, and rapid development time [40]. mRNA vaccine technology has multiple advantages [41]:

(i) compared to DNA vaccines, mRNA vaccines have no confirmed mechanism by which they would permanently modify the DNA of the affected cells. They are thus less prone to host genome integration and anti-vector immunity (a compromising factor of viral vectors) [42], with no potential for integration into host genome [43];

(ii) compared to live attenuated virus vaccines, there is no risk of infection following vaccination. Mutation of the poliovirus from the attenuated form in the vaccine to a virulent form is well known [44];

(iii) the efficiency is at least theoretically higher than in the case of adenoviral vectors that are already familiar to the human immune system, which sometimes neutralizes the adenovirus vector before exerting its effect [45];

(iv) unlike all living, attenuated or genetically modified viral vectors, whose production is rather difficult, mRNA can be synthesized rapidly and does not require cell cultures to be harvested [46].

Overall, mRNA vaccines have the potential to change how diseases can be prevented or treated. It is very likely that we are at a historical crossroads from a medical and pharmacotherapeutic point of view when we begin to replace traditional treatments with very complex and sophisticated immunotherapies. Engineering mRNA vaccines is based on the central dogma of molecular biology according to which RNA is converted to proteins using the protein translational machinery of the host. It is theoretically implausible for the new generation of mRNA vaccines to influence the source genetic code by integrating into DNA of the host genome. The following reasons support this claim [47]:

(i) no mechanism is known by which mRNA could be transported to or reach the nucleus, but it remains in the cytoplasm where it will be degraded after the translation step, and

(ii) even if it did arrive, it would not be able to interact with the DNA because the mRNA would have to be converted back to DNA.

### 2.3. Lipid Nanoparticles to Encapsulate the mRNA

The preference of the injected material for certain cells, tissues or organs depends exclusively on the biochemical characteristics of the LNPs where mRNA is encapsulated to avoid degradation by RNases and enable transfection of host cells. Nevertheless, the presence of ApoE facilitates the uptake of LNPs by immune cells, including monocytes and dendritic cells because these cells have shown efficient LNP uptake and mRNA translation at the injection sites and in the draining lymph nodes. Very small differences in the lipid composition of LNPs can cause huge differences in how they enter cells and which cells they enter. These small differences are mainly due to the chemical structure of lipids, such as cationic ionizable lipids, new generations of ionizable aminolipids, phospholipids or modified dendrimers [48].

According to a non-COVID-19 related study in monkeys, mRNA gradually leaves the injection site (hours, days) and locates in nearby lymph nodes where it can effectively trigger the immune cascade that leads to the production of antibodies [49]. From the anatomical point of view, if the vaccine is injected in the upper arm (deltoid muscle), it would be expected that most of the particles in the vaccine would be drained into the axillary lymph nodes but mRNA would extend beyond the lymph nodes [29]. This phenomenon very likely explains why common local reaction to the vaccine is swelling and axillary lymphadenopathy [50]. In other words, due to their short lifespan, vaccine-contained mRNA is unable of self-replication and in turn, it undergoes rapid RNA degradation by both extracellular and intracellular RNases [51]. Accordingly, the nucleoside modifications of mRNA vaccines not only enhance the stability of RNA but also reduce the innate immune response [52].

LNPs in the two COVID-19 mRNA vaccines (i.e., Pfizer/BioNTech and Moderna) have a particular composition which makes their distribution in the body particular. LNPs usually contain four ingredients: cholesterol as a membrane fluidity modulator/stabilizer, phospholipids to form a lipid bilayer structure, a lipid-linked polyethylene glycol (PEG) that helps prolonging the half-life of the composition (ALC-0315 and SM-102 are the pegylated lipids added by Pfizer/BioNTech and Moderna, respectively [53]), and ionizable cationic lipids whose positive charges bind to the negatively charged backbone of mRNA, thus improving the release of mRNA from the endosome to the cytoplasm [54].

Formulas that included PEG-2000 (such as COVID mRNA vaccines) may or may not [55] reach liver cells, depending on the molar concentration of PEG and the number of carbon atoms in the compound [56]. Moreover, the electrical charge of LNPs confered by the chemical composition can influence, e.g., their distribution to the lungs or spleen [57], the latter being another organ of great importance in the immune response. However, as the pH of normal arterial blood is ~7.4, the lipids are not charged, thus reducing cationic charge-mediated toxicity [58]. Given the vascularization of the skeletal muscles, a large part of the LNP-containing mRNA will be taken directly into the bloodstream and distributed in virtually any organ [59]. According to the assessment reports issued by the European Medicines Agency (EMA) for mRNA vaccines, only Pfizer/BioNTech conducted a nonclinical pharmacokinetic study with the same platform further used for clinical administration. Over 48 h, distribution was mainly observed in liver (major site of distribution), adrenal glands, spleen and ovaries, reaching maximum concentrations 8–48 h after injection. Concentrations higher that those found in plasma have been reported for the injection site, local lymph nodes, the spleen and eye [60].

### 2.4. Pharmacological Mechanism of Action of mRNA Vaccines

**Figure 2:**
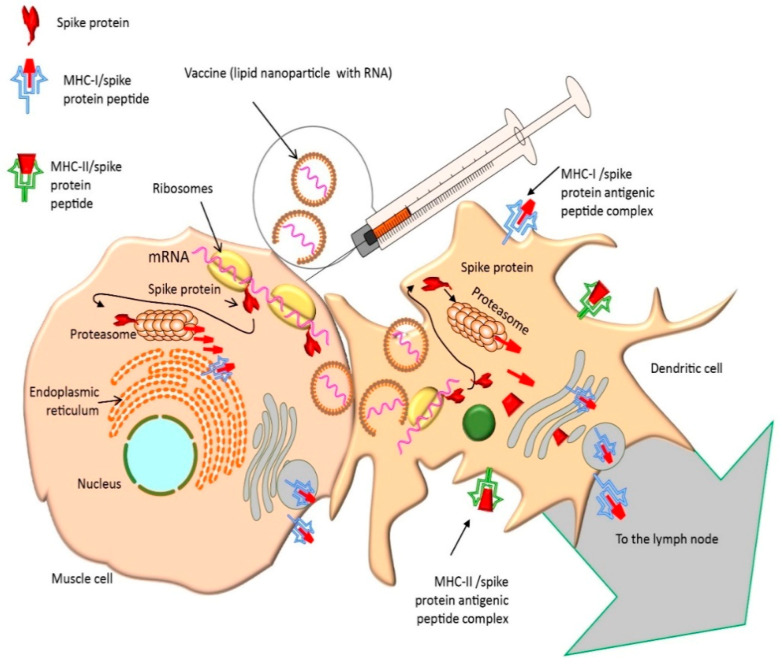
The pharmacological mechanism of action of intramuscularly administered mRNA vaccines (I). The vaccine releases LNPs containing mRNA encoding S protein into the muscle cells. However, most of the LNPs are uptaken by dendritic cells which translate mRNA into protein, process and present the protein peptides binding them to MHC-I and MHC-II to naïve B and T cells in the regional lymph nodes. Legend: messenger RNA (mRNA), lipid nanoparticles (LNPs), major histocompatibility complex (MHC).

**Figure 3:**
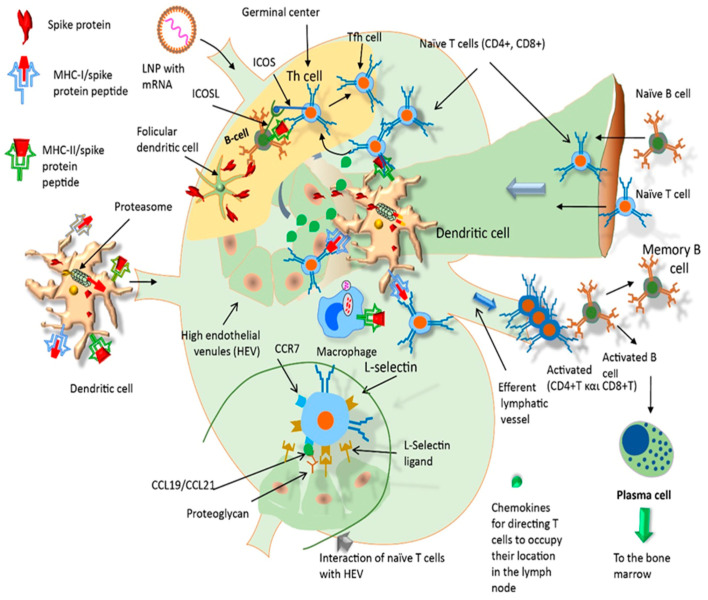
The pharmacological mechanism of action of intramuscularly administered mRNA vaccines (I). Dendritic cells travel through the lymphatic vessels and enter the lymph node by afferent lymph vessels. In addition, LNPs alone also enter the lymph node by afferent lymphatics, and they are uptaken by native macrophages of the lymph node. On the other hand, naïve B and T cells enter the lymph node by arterioles which inside the lymph node shift to high endothelial venules and provide ligands to transiently stop the flow of these cells which then, guided by chemokines, move into the lymph node. Dendritic cells and native macrophages of the lymph node process and present appropriately S protein-peptide fragments to naïve CD4+T, CD8+T and B cells which become armed effector T lymphocytes and plasma cells, respectively. Legend: inducible T cell costimulator (ICOS), ICOS ligand (ICOSL), high endothelial venules (HEV), chemokines (CCL), cluster differentiation (CD).

After intramuscular injection of mRNA vaccines, the released LNP-containing mRNA reach deeper tissue and can transfect muscle cells through endocytosis [61]. It is possible that mRNA will be translated to S protein inside the muscle cells. Shortly thereafter, antigen presenting cells (APCs, particularly dendritic cells and macrophages) are recruited to the site of injection such that most of the LNP containing mRNA is taking up by these cells. APCs then migrate to the lymph node nearest to the vaccination site (Figure 2). Passive drainage of mRNA–LNPs through lymphatic vessels allows for direct delivery of mRNA to the lymph nodes containing resident APCs within them, e.g., subcapsular sinus macrophages [62] (Figure 3).

Thanks to its lipid structure, endocytosis of LNPs occurs and the mRNA is released into the host cell cytosol, where mRNA combines with ribosomes and begins to be translated to form metastable trimeric prefusion S protein. After translation, the S protein is shuttled through the endoplasmic reticulum and Golgi complex resulting in the presentation of the full length S protein assembled in a trimer structure at the cell membrane [63]. This membrane antigen (not in the context of major histocompatibility complex –MHC–) is the main target for B cell recognition [64]. Although the full length S protein is translated and then transported to the plasma membrane, the majority of the protein is ubiquitinated and enters into the proteasome where it is cleavaged to antigenic peptides. The S protein, or fragments thereof, bind to newly synthesized MHC Class I (MHC-I) molecules and then are displayed on the cell surface (Figure 2).

On the other hand, APCs present processed antigens to T cytotoxic cells (CD8+) and T helper cells (CD4+) via binding to MHC class I or II molecules, respectively, thus triggering cellular immunity. T helper cells also play a key role on B cell priming for antibody responses [32]. Vaccine-driven production of type I interferon promotes differentiation of CD4+ and CD8+ effector T cells producing inflammatory and cytotoxic mediators, and CD4+ T follicular helper (TFH) cells, which promote B cell differentiation into antibody-secreting plasma cells [65] (Figure 3). Type I interferon has been shown to amplify T cell memory and promote B cell differentiation and survival, suggesting that vaccine-associated inflammation in the booster can further promote generation and perpetuation of long-term immunological memory. Both specific antibodies and T cell cytotoxicity kill APCs showing the S protein, or antigenic fragments thereof, on their plasma membrane through the MHC-2 complex. Because the overall immune reaction is specific for the SARS-CoV-2 virus, the immune system is primed to protect against future infection.

## 3. Duration of the Vaccine Protective Effect: Approaches and Challenges

Historically, vaccines have almost always produced weaker or shorter immunity compared to natural infection [66]. Therefore, depending on what the long-term studies for this generation of mRNA vaccines will show, there may be a need for boosters every two to three years to maintain an effective immune response. Many vaccines require a booster at a certain age, for example, that for hepatitis B [67].

Nonetheless, determining serum antibodies is only an approximation of the body’s ability to neutralize SARS-CoV-2. For example, their presence does not always guarantee protection, there are still ambiguities about which IgG isotypes are truly effective [68]. Conversely, it is not yet known whether the absence of antibodies to those immunized by disease or vaccine necessarily means the absence of protection. In other words, we do not know whether or not B lymphocytes are capable of rapid reactivation upon new contact with the virus. Moreover, the absence of antibodies does not mean that we do not have other resources to fight the infection effectively; the cellular immune response, through T lymphocytes, plays a crucial role in COVID-19 [69].

There are several types of T lymphocytes: Cytotoxic T lymphocytes (CD8+) have the ability to recognize virus-infected or transfected cells such as those presenting the mRNA vaccine-induced S protein fragments and destroy these cells by direct contact. Helper T lymphocytes (CD4+) secrete cytokines and regulate the activity of B lymphocytes and cytotoxic T lymphocytes [70]. Regulatory CD4+ T lymphocytes are crucial for the maintenance of immunological tolerance. Their major role is to shut down T-cell-mediated immunity toward the end of an immune reaction and therefore protect the organism from an uncontrolled immune response because they suppress activation, proliferation and cytokine production of CD4+ T cells and CD8+ T cells and are thought to suppress B cells and dendritic cells [70]. Regulatory T cells can produce soluble messengers which have a suppressive function, including transforming growth factor beta (TGF-β), IL-10 and adenosine. These cells are characterised by the co-expression of CD4 T cell co-receptor and CD25, which is a component of the IL-2 receptor. Both types of lymphocytes (B and T) can become memory lymphocytes, able to recognize a certain antigen (e.g., foreign protein) in the long-term [71]. Unlike the humoral immune response, memory T lymphocytes appear more durable after SARS infections. For example, six years after SARS-CoV infection, recovered patients had no trace of antibody, but 61% of them still had T lymphocytes capable of recognizing SARS proteins [72]. Another study found that reactivity was maintained at 17 years after SARS infection [73].

In the case of SARS-CoV-2 infection, a robust CD4+ and CD8+ T lymphocyte memory is induced. In particular, CD4+ cells are still present in 92% of recovered patients beyond six months after infection, and it is estimated that after an initial decrease, a relatively stable plateau is reached, in which these cells no longer decrease [74]. Part of the population that has never come in contact with the virus may show CD4+ and CD8+ T lymphocytes able to recognize SARS-CoV-2 [75]. The explanation is that there is a high degree of cross-reactivity between these cells and common coronaviruses responsible for seasonal colds (OC43, 229E, NL63 and HKU1). Indeed, this could represent one of the reasons why there are differences in the severity of the disease between individuals [76]. Uncertainties about the true effectiveness of the measured anti-SARS-CoV-2 antibodies are probably the main reason why vaccination is recommended even in people who have undergone the disease and still have detectable IgG [77]. The recommendation to vaccinate people with past SARS-CoV-2 infection is also based on the increase in IgG antibodies upon vaccination. Distinct antibody and memory B cell responses in SARS-CoV-2 naïve and recovered individuals following mRNA vaccination has been reported [78]. In fact, the recommendation to vaccinate people with a previous SARS-CoV-2 infection is also based on an increase in IgG antibodies to vaccination. The generation of memory mediated by protective antibodies is a germinal reaction and occurs in the case of SARS-CoV-2 infection as well as in the case of vaccination [79].

More recently, Tarke et al. (2021) have shown that people recovered from COVID-19 or who have received Pfizer-BioNTech or Moderna vaccines maintain a ‘second line of defense’ capable of recognizing the variants of concern of the SARS-CoV-2 because T cells (both CD4+ and CD8+) continue to recognize mutated forms of the virus [80]. The T cell response may thus contribute to limiting COVID-19 severity induced by variants of concern (i.e., United Kingdom, South Africa, Brazil and California) that partially or largely escape neutralizing antibodies

## 4. Potential Secondary Reactions to mRNA Vaccines

Unlike inactivated and recombinant protein vaccines, mRNA vaccines do not need adjuvants to enhance the magnitude and duration of the immune response. LPNs containing mRNA and the mRNA itself are already immunogenic enough to induce an effective immune response.

Adjuvants increase local inflammation, i.e., by amplifying the recruitment of immune cells, which speeds up the generation of antibodies [81]. This immunogenic character is responsible for rare cases of severe allergic reactions. There is a possibility that delayed hypersensitivity reactions may occur after vaccination, with clinical symptoms such as large localized swelling or nodules on the surface of the skin. These reactions are considered to be mediated by immune cells such as T lymphocytes and have been attributed to some excipients and have not been considered as a contra-indication for subsequent vaccination [81]. The administration of the vaccine is done in settings, which have the resources to treat anaphylactic shock quickly and effectively [81].

Following intramuscular injection of mRNA vaccines, LNPs are expected to circulate in the bloodstream; however, owing to the relatively low antigenity of the contents, severe allergic reactions are not to be expected [82]. After widespread use of COVID-19 mRNA vaccines, severe allergic (anaphylactic) reactions to mRNA vaccines are very rare and have been estimated at 4.2 cases per million doses, with a relative incidence of anaphylaxis of two and seven times higher for individuals having a prior history of allergies and/or anaphylaxis, respectively [83]. According to the UK Medicines and Healthcare products Regulatory Agency (MHRA), 29 million doses of Pfizer/BioNTech vaccines have been administered in the UK as of 23 June 2021 and a total of 413 anaphylactic/anaphylactoid responses (reactions or shock) were reported (two of them fatal), which represents an incidence rate of 14.2 cases per million doses. As for the Moderna vaccine, only 0.88 million doses were administered, and 21 cases were reported (none of them fatal), representing 23.9 cases per million doses.

These allergies are not due to the mRNA itself because the RNA contains modified N1-methyl pseudouridine instead of uridine to minimize the immune responses and cytotoxicity induced by introducing mRNA into cells [84]. However, RNA trace impurities present in mRNA vaccines might result in expression of aberrant proteins that could trigger delayed immunological reactions in some subjects [84].

Conversely, LNPs may represent a possible source of allergens because anaphylactic reactions have been reported in some recipients of intravenously infused PEGylated nanomedicines [85]. LNP pegylation is used to reduce the immunostimulatory potential of the nanopartices, thus contributing to an improved safety and to a reduced clearance by the mononuclear phagocyte system [86]. Among them, most likely PEG is a nontoxic but known allergen, widely used in shampoos, toothpaste and laxatives.

Despite of the potential risk of anaphylaxia, regulatory agencies from the European Union (EMA) and Unites States (FDA) consider that vaccinations are contraindicated only in case of allergy to one of the vaccine components or if there was a severe allergic reaction to the first dose.

## 5. Autoimmune Diseases and COVID-19: A Possible Tie-Up

Triggers of autoimmune disease are still incompletely elucidated, but at least in theory, any disruption of the immune system can cause an autoimmune syndrome in people who have a particular genetic predisposition, or an exacerbation (crisis or episode) in those who have the disease already [76,87]. Because we know so little about the occurrence of these diseases, the possibility that, under certain circumstances, a vaccine (perhaps including the mRNA ones) may trigger an autoimmune reaction cannot be ruled out.

Although the potential association between mRNA vaccine and autoimmunity has not yet been addressed, no confirmed cases of triggering or worsening of autoimmune diseases have been reported so far. Indeed, patients with pre-existing autoimmune diseases were excluded from phase 3 studies with mRNA vaccines, so such information is missing and potential autoimmune events will be monitored in the post-authorisation phase.

Although the association between vaccines and autoimmune diseases is very weak or non-existent in case of the mRNA ones, the association of these diseases with viral infections is very well documented. [87]. Viruses, in particular, are known to trigger autoimmune and/or autoinflammatory conditions, and SARS-CoV-2 is no exception, as a significant number of cases of autoimmune diseases have been triggered by COVID-19. Therefore, vaccination should also be recommended for people with autoimmune diseases if they have no other contra-indication [88].

While there are clear data that SARS-CoV-2 can trigger autoimmune diseases, there are no data pointing out mRNA vaccines as a trigger. Nevertheless, mRNA vaccines have been hypothesized to bind pattern recognition receptors (PRRs) in endosomes or cytoplasm, or can be recognized by Toll-like receptors (TLR7 and TLR8), eventually resulting in activation of several pro-inflammatory cascades, such as the assembly of inflammasome platforms, the type I interferon response and the nuclear translocation of the transcription factor nuclear factor (NF)-kB. Upregulation of these immunological pathways also occurs in immune-mediated diseases, especially in genetically predisposed individuals [87].

## 6. Future Perspectives and Overall Conclusions

Taken together, the medical and technological challenge of developing and delivering highly-effective vaccines based on mRNA technology in less than one year, when the average vaccine development is over 10 years [89], shows that a very powerful platform technology has been utilized. The potential application of mRNA vaccines for other infectious diseases and other medical conditions is only emerging. The tighter control of the manufacturing process overcomes the limitation of traditional efficacy testing and of safety and batch-release tests, which contributes to the traditionally long development cycles [90]. However, a comprehensive international program is needed to study the molecular interactions of RNA vaccines as well as other recombinant vaccines with immune system cells because there is a risk that the balance between Th1 and Th2 will be affected and will dominate Th1, thus increasing the risk of triggering an acute episode of a pre-existing autoimmune disease [89].

Evaluation of vaccine safety and efficacy in immunocompromised individuals is challenging, given that new therapies are emerging for which information on vaccine safety and efficacy is not yet fully analyzed. The general principles in the evaluation of vaccination in adult immunodeficiencies pay additional attention to the evaluation of the immune status; risk assessment in relation to benefits; the existence of known recommendations for current vaccination; administration of vaccines before the onset of immunosuppression if possible; and antibody testing to measure the post-vaccination immune response [91].

One of the limitations of this manuscript is the lack of presentation of final data on the efficacy of mRNA vaccines in the Delta variant of SARS-CoV-2. No vaccine fully protects against Delta-type infections, but the low rate of hospitalizations so far shows that, at least in this regard, vaccines prevent the development of new severe forms of COVID-19.

mRNA vaccines are also being investigated for the prevention of several infectious diseases, such as rabies, influenza, Zika, Ebola, HIV infection, respiratory syncytial virus infection, hepatitis C virus, but none have yet reached an advanced stage of a clinical study. The production of a safe and effective influenza mRNA vaccine is an important target for the development of this technique because the process currently used to obtain influenza vaccines is slow and laborious, but it can be significantly accelerated by the use of mRNA technology.

Many candidate vaccines using mRNA technology are currently under development for the prevention and, especially, the treatment of cancer. They encode tumour-associated antigens stimulating cell-type immune response in order to eliminate or inhibit malignant cells. Some of these vaccines have reached clinical trials and address neoplastic processes such as melanoma, breast cancer, acute myeloid leukaemia, multiple myeloma, mesothelioma, glioblastoma, renal cell carcinoma, pancreatic cancer, nonmicrocellular lung cancer, prostate cancer, glioma, ovarian cancer, etc. Another potential use of mRNA vaccines is immunoprophylaxis and passive immunotherapy (for example, in the case of infections such as HIV, cytomegalovirus, HPV, etc.), through the use of mRNA molecules encoding light or heavy chains of antibodies (immunoglobulins).

An important point for registered vaccines is represented by regulatory requirements for the production of mRNA vaccines because requirements for their use have not been developed. There are no reference drugs for comparison. New mechanisms of behavior of mRNA vaccines in the human body are possible but were not known until now (for example, many molecular mechanisms were discovered with HIV viruses). It will be important to know the impact of these mechanisms on various groups of people, e.g., the effect of mRNA vaccines on blood clotting in patients with immunodeficiency and oncology.

## Data Availability

The dataset presented in this study is available from the corresponding author upon reasonable request.
